# Supplementary material for: Benchmarking Hi-C scaffolders using reference genomes and de novo assemblies
Source: Genome Biol. 2026 Mar 10;27:133. doi: 10.1186/s13059-026-03978-3 (PMC13085270; doi:10.1186/s13059-026-03978-3)
Supplement: Supplementary file 1 — Additional file 1: Additional supplementary figures detailing our literature search, genome scaffolding results, data sources, and additional visualizations. [file 13059_2026_3978_MOESM1_ESM.docx]

**Supplement**
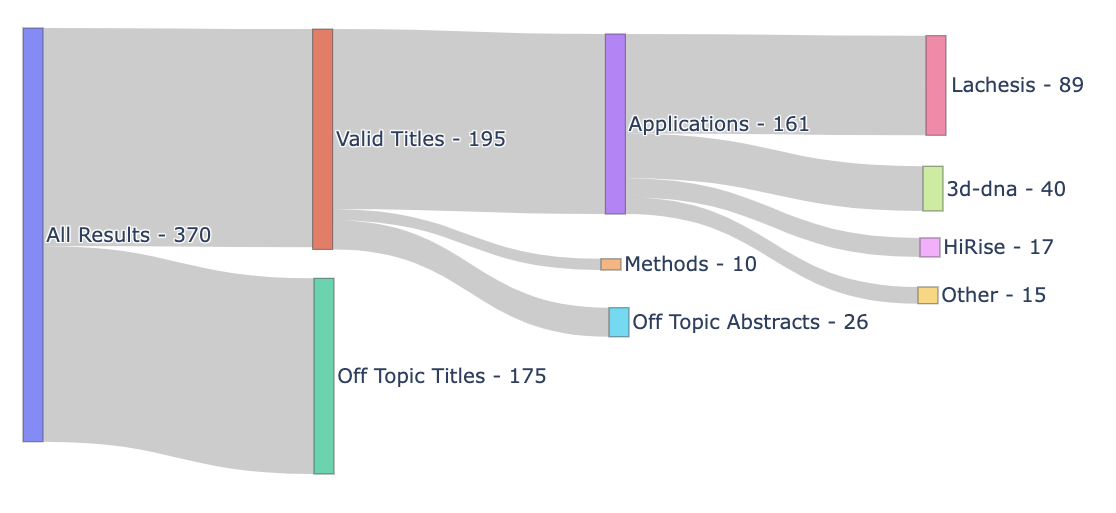


**Fig. S1.** A sankey diagram depicting the literature search process. We identified ten different Hi-C scaffolding methods in the literature and found that their usage varied significantly, with only five methods showcasing more than three published genomes.

|  | **Grouping** | | **Order** | | **Orientation** | | **Accuracy** | |
| --- | --- | --- | --- | --- | --- | --- | --- | --- |
| **Method** | **Split** | **De novo** | **Split** | **De novo** | **Split** | **De novo** | **Split** | **De novo** |
| lachesis | **0.91 ± 0.1** | **0.71 ± 0.2** | **0.95 ± 0.03** | **0.66 ± 0.1** | **0.95 ± 0.03** | **0.55 ± 0.2** | **0.95 ± 0.03** | 0.55 ± 0.2 |
| hirise | 0.74 ± 0.3 | 0.69 ± 0.2 | 0.87 ± 0.2 | 0.49 ± 0.3 | 0.87 ± 0.2 | 0.44 ± 0.3 | 0.87 ± 0.2 | **0.62 ± 0.2** |
| 3d_dna | 0.35 ± 0.2 | 0.38 ± 0.3 | 0.66 ± 0.3 | 0.34 ± 0.2 | 0.66 ± 0.3 | 0.30 ± 0.2 | 0.71 ± 0.2 | 0.41 ± 0.2 |
| salsa | 0.52 ± 0.3 | 0.47 ± 0.3 | 0.85 ± 0.2 | 0.44 ± 0.2 | 0.84 ± 0.2 | 0.41 ± 0.2 | 0.85 ± 0.1 | 0.50 ± 0.2 |
| allhic | 0.75 ± 0.4 | 0.52 ± 0.3 | 0.95 ± 0.1 | 0.58 ± 0.3 | 0.94 ± 0.1 | 0.49 ± 0.3 | 0.84 ± 0.2 | 0.39 ± 0.3 |
| baseline | 0.18 ± 0.3 | 0.36 ± 0.3 | 0.03 ± 0.1 | 0 | 0.03 ± 0.1 | 0 | 0.16 ± 0.2 | 0.28 ± 0.3 |

**Table S1.** An overview of performance of each of the methods based on their average accuracy determined by Edison. The split column refers to the task of scaffolding equal sized pieces of the reference genome. The *de novo* column refers to the task of scaffolding the assemblies created by Canu. Baseline represents the score for contigs without scaffolding.

| **Organism** | **Genome Size** | **Reads** | **Bases** | **Coverage** | **BioProject** |
| --- | --- | --- | --- | --- | --- |
| S. cerevisiae | 12,100,000 | 313,114 | 1,701,530,052 | 141 | PRJEB7245 |
| L. tarentolae | 32,200,000 | 1,360,815 | 7,198,339,498 | 224 | PRJNA821548 |
| A. thaliana | 135,000,000 | 7,353,356 | 49,942,606,909 | 370 | PRJNA314706 |
| H. sapiens | 3,100,000,000 | 47,885,330 | 328,978,598,683 | 106 | PRJNA301527 |

**Table S2.** Overview of data collected for *de novo* genome assemblies. The amount of data is roughly proportional to the size of the genome such that they can be down-sampled to a similar read coverage.

| **Coverage** | **Yeast N50** | **Leishmania N50** | **Arabidopsis N50** | **Human N50** |
| --- | --- | --- | --- | --- |
| 10 | 21,825 | 9,250 | 24,823 | 18,711 |
| 20 | 176,516 | 18,595 | 86,731 | 43,231 |
| 30 | 551,752 | 41,537 | 159,879 | 105,563 |
| 40 | 568,123 | 40,610 | 147,845 | 626,886 |
| 50 | 614,056 | 94,577 | 149,418 | 1,873,143 |
| 60 | 813,309 | 213,275 | 161,138 | 2,018,914 |
| 70 | 777,771 | 71,335 | 140,209 | 4,761,131 |
| 80 | 813,629 | 117,224 | 149,909 | 7,508,518 |
| 90 | 813,427 | 229,090 | 164,531 | 9,231,632 |
| 100 | 930,538 | 332,478 | 186,445 | 10,553,285 |

**Table S3.** Overview of the *de novo* assemblies created by Canu. We generated ten assemblies for each species and downsampled reads used to create them to vary their N50s. As a general trend, we observed that increased read coverage led to long contigs. Arabidopsis appeared to be an outlier of this trend, and its genome assembly appeared to be challenging and indicative of high rates of heterozygosity.


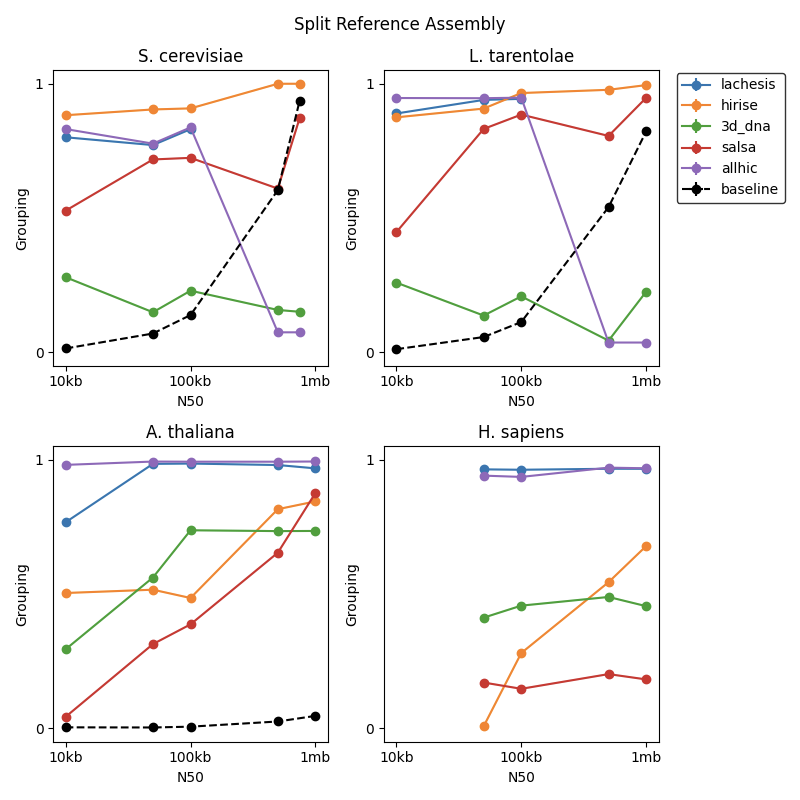


**Fig. S2.** The grouping scores of scaffolders on split reference assemblies. There is wide variation in grouping performance, with trends pointing to difficulty with small assemblies with large N50s and large assemblies with small N50s.


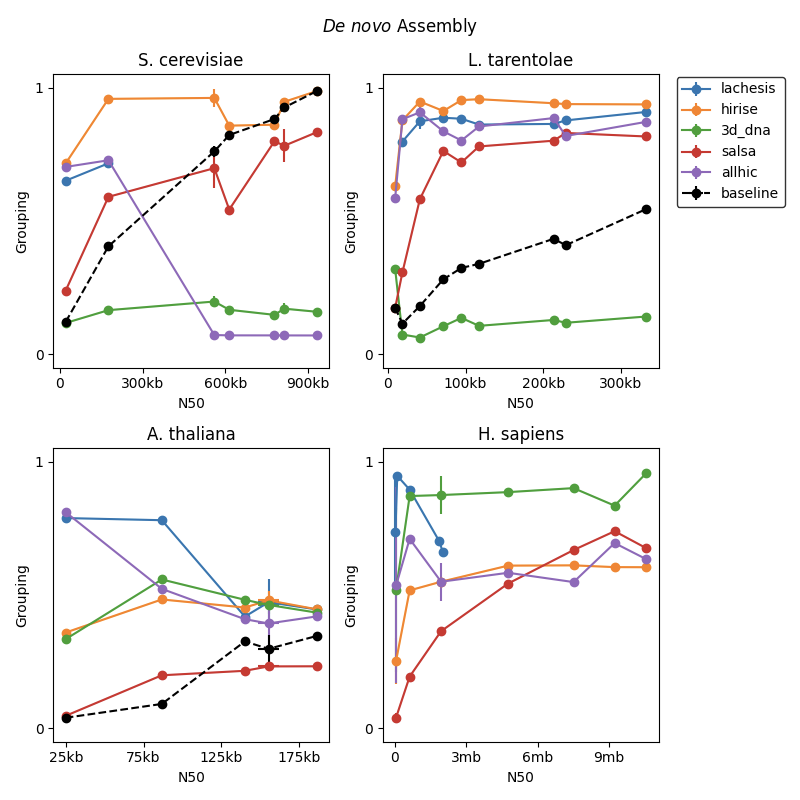


**Fig. S3.** The grouping scores for Hi-C scaffolders on *de novo* assemblies. Higher grouping accuracy indicates that scaffolders were able to uniquely isolate contigs belonging to the same chromosome within scaffolds.


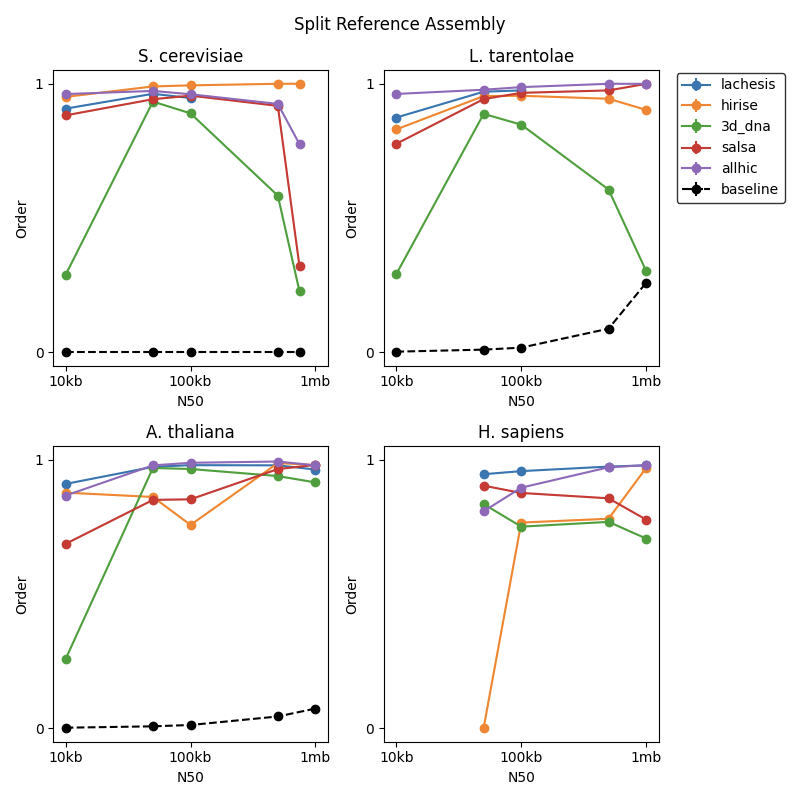


**Fig.S4.** The order scores for Hi-C scaffolders on split reference assemblies.


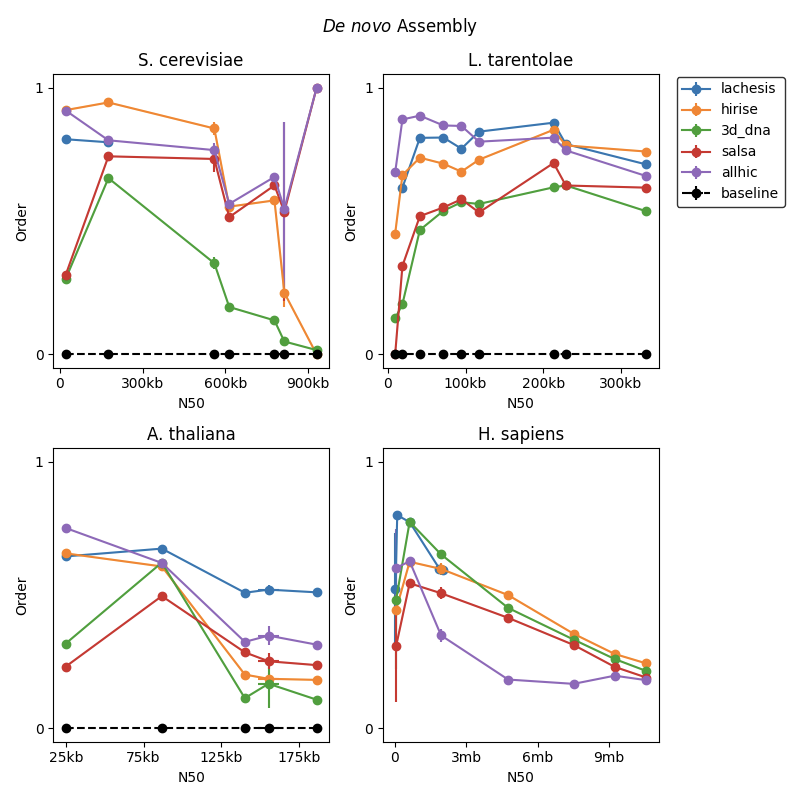


**Fig. S5.** The order scores for Hi-C scaffolders on *de novo* assemblies. Higher order accuracy indicates that scaffolders were able to correctly place contigs next to their expected neighbors.


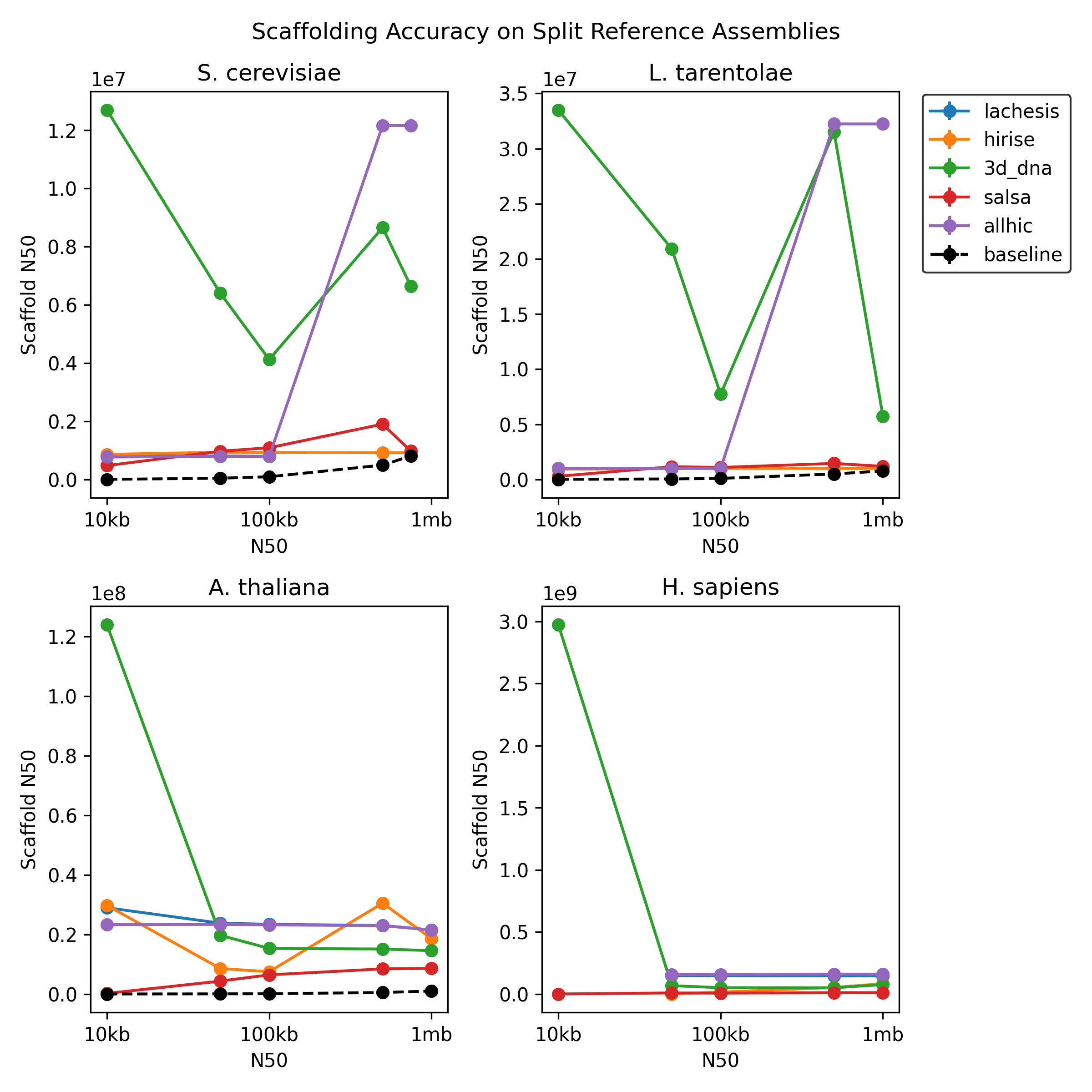


**Fig. S6:** The scaffolding N50 vs the assembly N50 for the split reference assemblies. We see that 3d-dna and AllHiC are prone to “over-scaffolding”, where a far greater than expected number of contigs are joined together, often exceeding even the N50 of the underlying chromosomes.


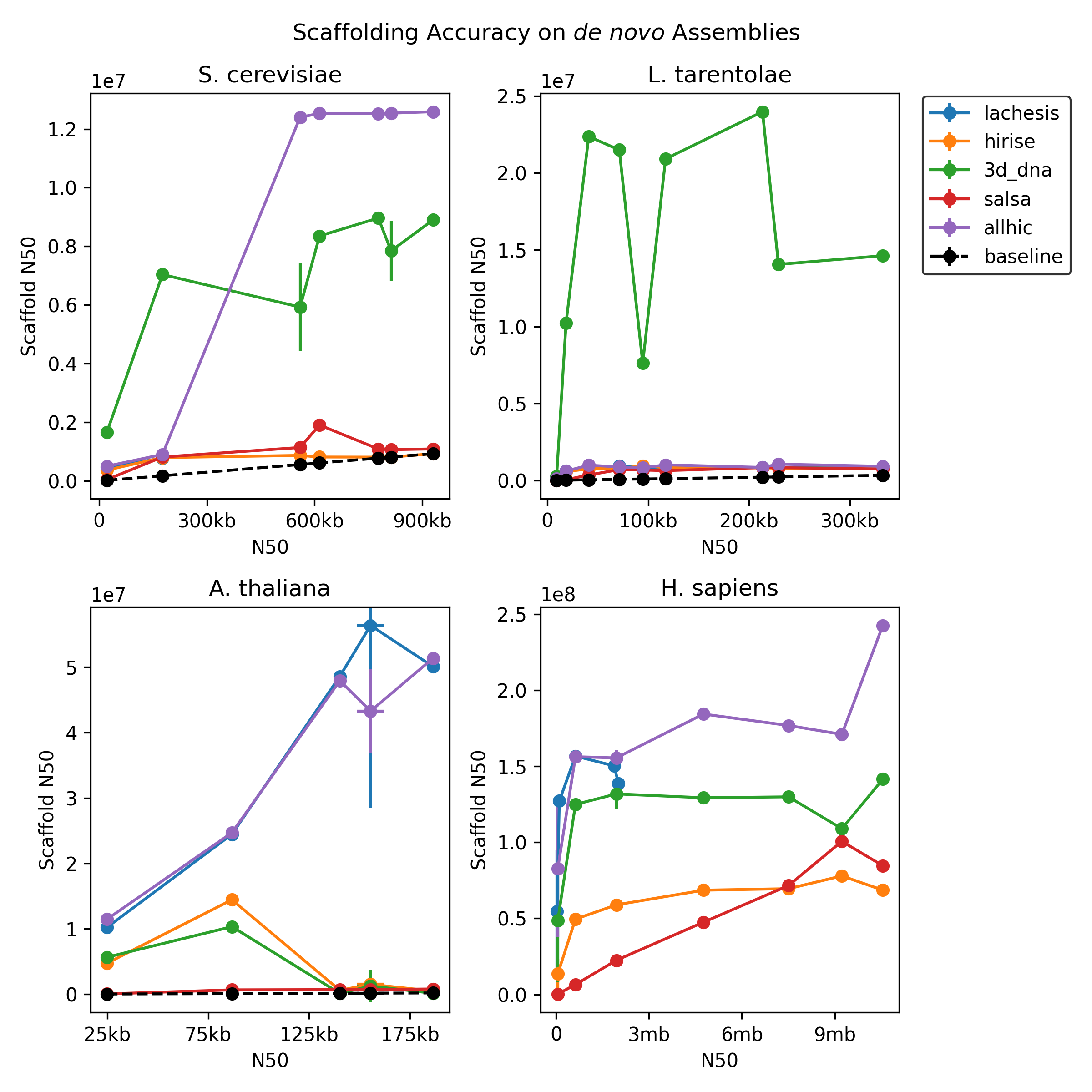


**Fig. S7:** The scaffolding N50 vs the assembly N50 for the *de novo* assemblies.


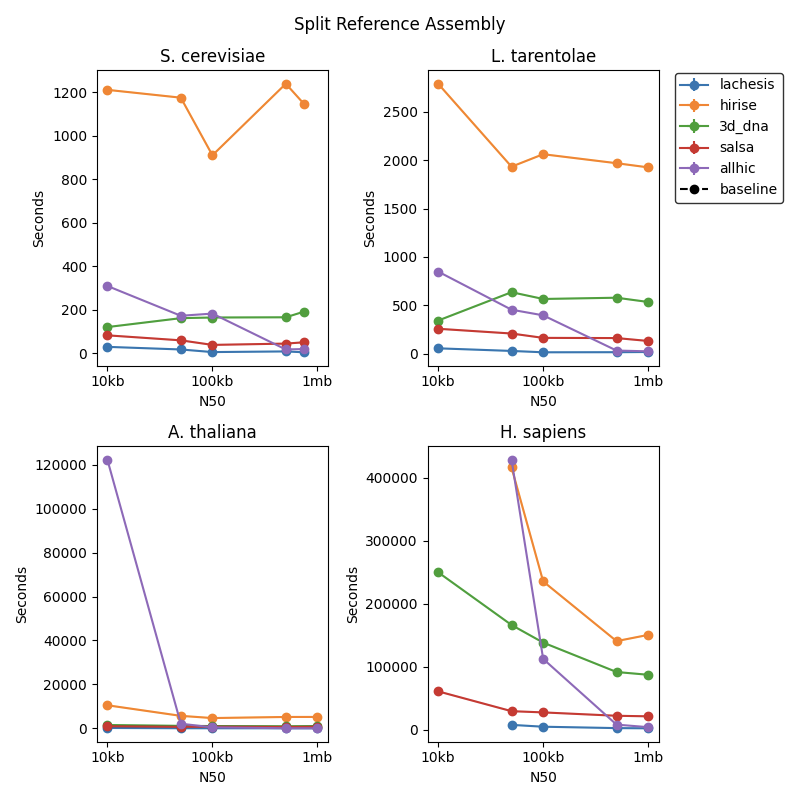


**Fig. S8**. The runtime of Hi-C scaffolders on split assemblies. The runtime refers to the amount of time it takes for a given scaffolding run to finish, given the same amount of computational resources.


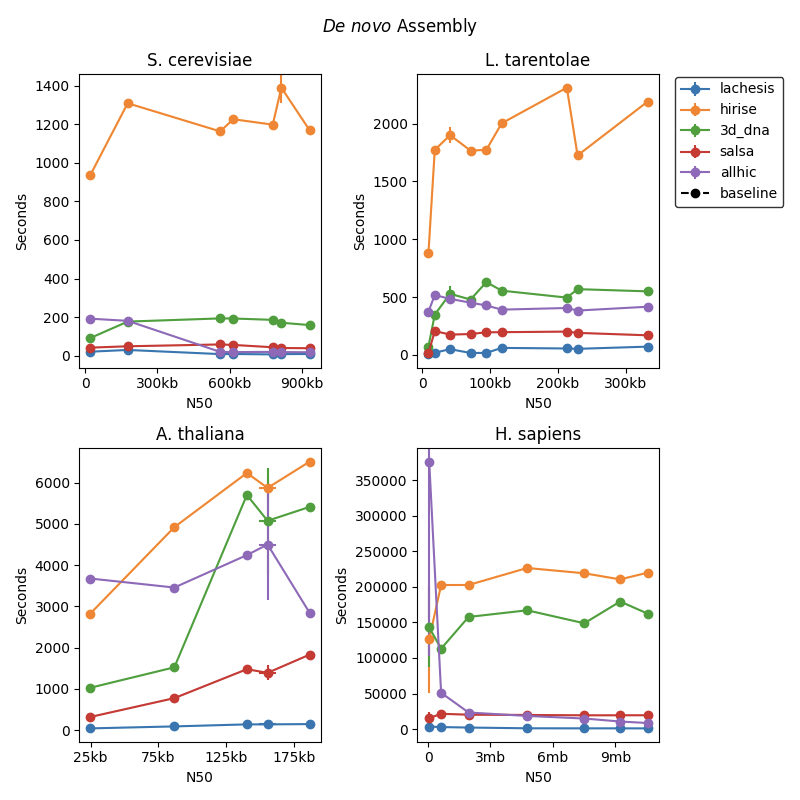


**Fig. S9.** The runtime of Hi-C scaffolders on *de novo* assemblies. Hirise is generally the slowest and Lachesis the fastest scaffolder.


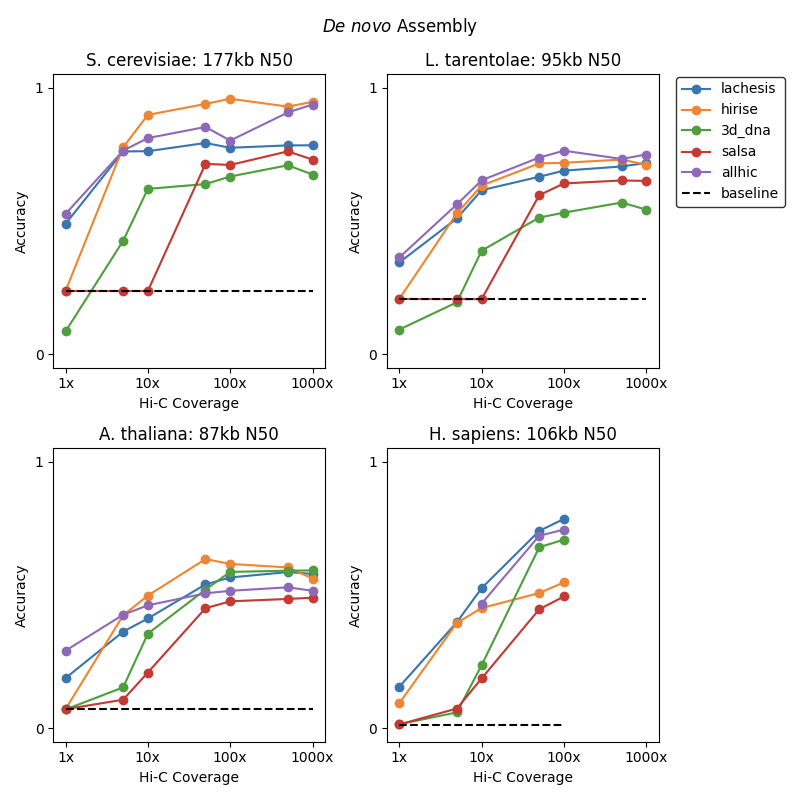


**Fig. S10.** Downsampling of Hi-C reads on *de novo* assemblies. The same trend as the split references is seen here, where Hi-C read densities below 50 reads per kilobase lead to a decline in performance.


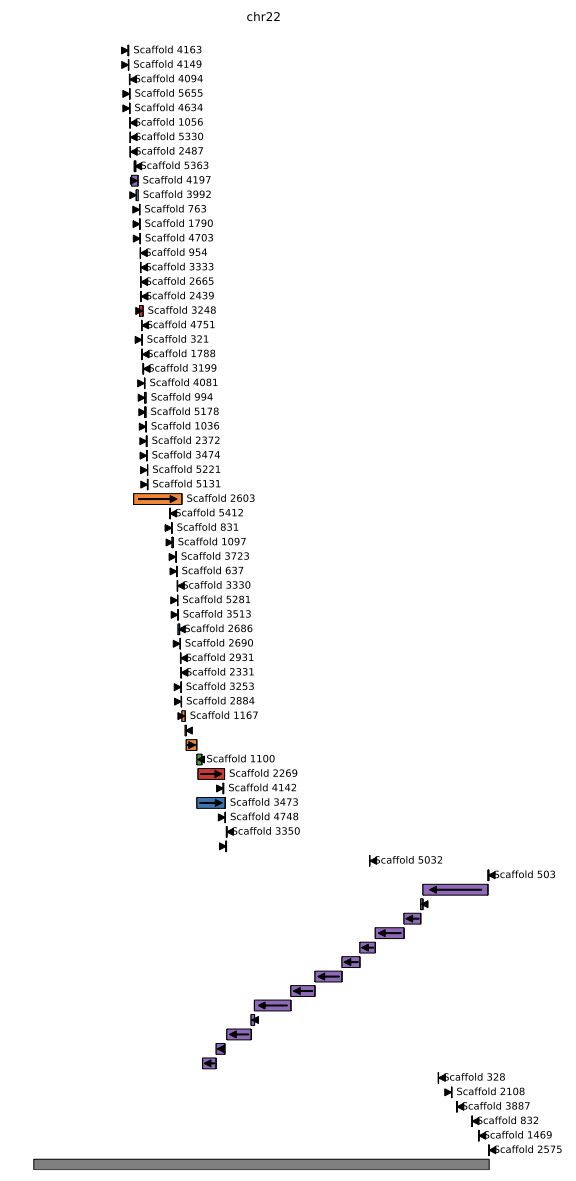


**Fig. S11.** An overview of how HiRise scaffolded the 10mb N50 *H. sapiens* assembly for Chromosome 22. Each row represents a contig, and each label and color represents a scaffold. The x-axis represents the alignment based position of the contig, and the y-axis represents the scaffolder based order of the contigs. Here, HiRise picks out a set of larger contigs and scaffolds them in the correct order relative to each other. However it leaves out a number of the smaller contigs, including ones that overlap with its primary scaffold (Scaffold 503) for this chromosome.


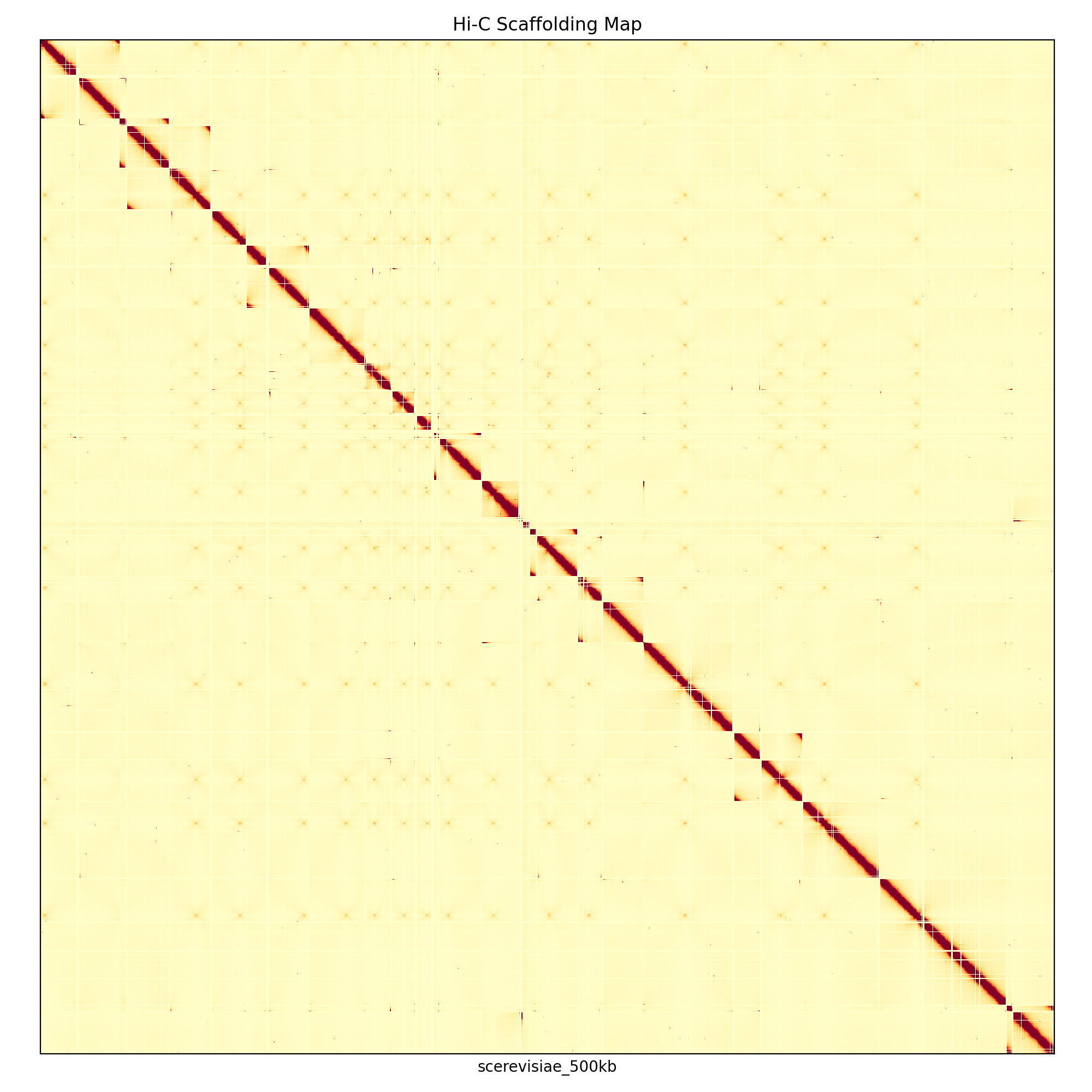


**Fig. S12.** AllHiC scaffolding 500kb contigs from the split reference assembly of *S*. *cerevisiae.* While the contigs have been placed mostly in the correct order and orientation, all the contigs were placed in a single mega-scaffold causing the overall accuracy to dramatically decrease for this particular scaffolding.


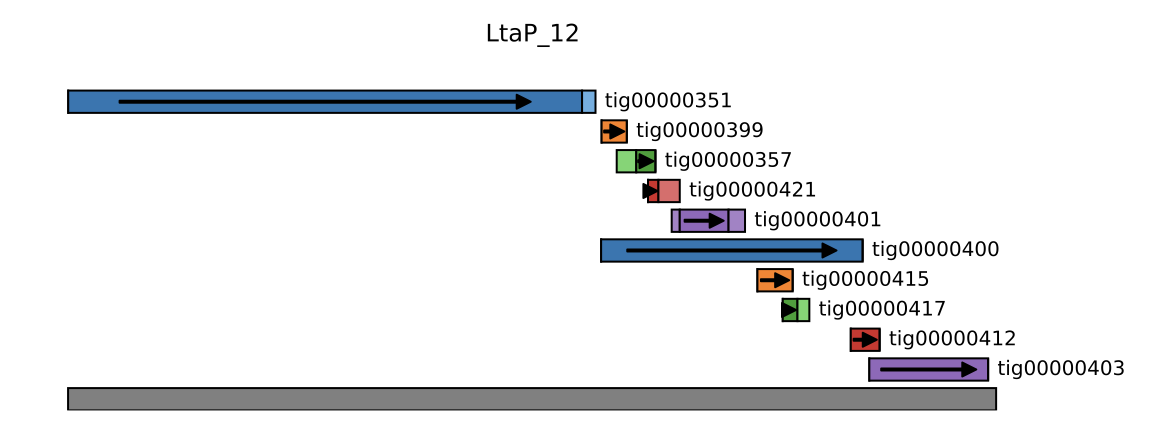


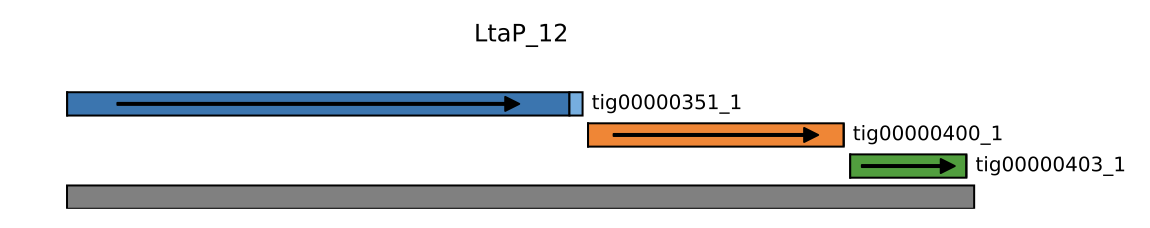


**Fig. S13.** Using purge_dups to remove halpotigs. The top section shows the contigs of the original assembly for L. tarentolae that map to chromosome 12. The bottom section shows the remaining contigs after the purging of haplotigs.


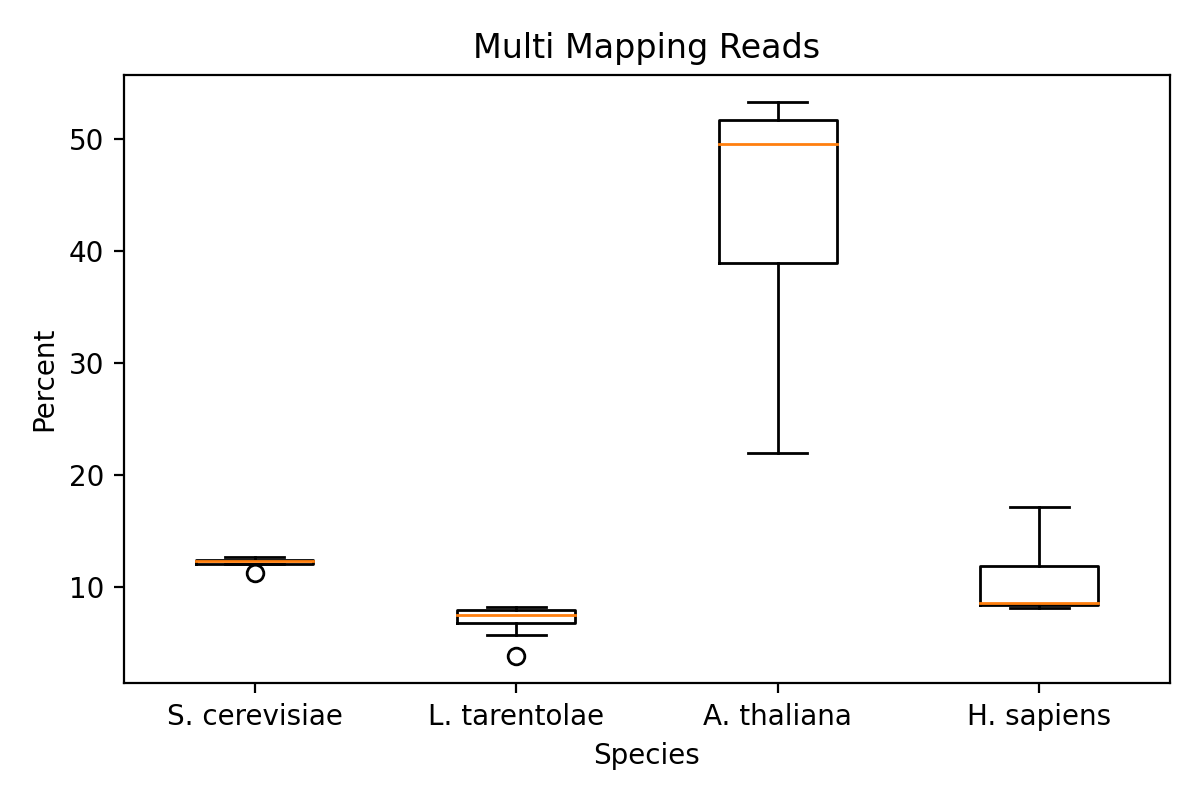


**Fig. S14.** The percent of reads that map to multiple positions in the *de novo* assembly. We found that as the number of reads used to create the *de novo* assembly goes up, the repetitive content of the genome goes up. The uniformly low accuracy against *A. thaliana* assemblies can likely be attributed to a high percentage of multi-mapping reads, which cannot be used by Hi-C scaffolders.
