# Supplementary material for: Benchmarking Hi-C scaffolders using reference genomes and de novo assemblies
Source: Genome Biol. 2026 Mar 10;27:133. doi: 10.1186/s13059-026-03978-3 (PMC13085270; doi:10.1186/s13059-026-03978-3)
Supplement: Supplementary file 2 — Additional file 2: Review history. [file 13059_2026_3978_MOESM2_ESM.docx]

Review History

**First round of review**

**Reviewer 1**

**Were you able to assess all statistics in the manuscript, including the appropriateness of statistical tests used?**

Yes, and I have assessed the statistics in my report.

**Comments to author:**

This manuscript deals with the method for genome scaffolding using Hi-C data. The authors compared the performance of five different computational programs developed for Hi-C scaffolding in four different species. The theme of the study is of great importance, but the authors could not soundly design the study. In short, the setting of using each program needs to be optimized for every single case, depending on the genome size of the species of interest, the sequence length distribution of input contig sequences, and the choice of the restriction enzymes used in Hi-C library preparation, and without this kind of optimization, performances of different programs cannot be compared, for the purpose of concluding which program performs better or worse.

Another caveat is that testing the program's scaffolding performance using split sequences into a uniform length is too far from realism. Normally, there is a huge variation of the continuity of input sequences, which is related to the distribution of difficult regions with high repeat density and GC content.

The authors used the 'known' existing reference genome assembly (without specifying them in particular) as a ground for quantifying the accuracy in scaffolding, but this is not justified as a single metric (although divided into orientation accuracy and ordering accuracy) for the benchmark, because the reference assemblies may contain mistakes.

To improve the entire design of the study, I recommend the authors to refer to the literature included below and more cited therein.

<https://genomebiology.biomedcentral.com/articles/10.1186/s13059-022-02765-0>
<https://onlinelibrary.wiley.com/doi/10.1111/mec.16146>
<https://www.nature.com/articles/s41586-021-03451-0>

**Reviewer 2**

**Were you able to assess all statistics in the manuscript, including the appropriateness of statistical tests used?**

Yes, and I have assessed the statistics in my report.

**Comments to author:**

Major comments:
1. The authors only tested scaffolding accuracy and speed of the scaffolders, however scaffolding speed is not very well explained in the text, while scaffold N50 is not measured.
2. The comparison among the scaffolders is not very reasonable, tools like AllHiC and Lachesis require a chromosome number as input, while other not. It's more appropriate to treat them as two groups and perform the comparision;
3. More scaffolders should be involved into this test to find the best performer.

Minor comments:
1. the authors should explain how different N50 assemblies are obtained.
2. The authors should list the genome accessions instead of project ID.
3. we recommend to use T2T assemblies to perform the test, since they should contain fewer errors.
4. The author should mention what coverage should be applied to get more accurate scaffolds for most scaffolders.

**Authors Response**

**Point-by-point responses to the reviewers’ comments:**

**Summary:** With the widespread adoption of Hi-C as an experimental method, we seek to benchmark the most widely used Hi-C scaffolders in the field. The reviewers provided valuable insight into the experimental design, point out various opportunities to strengthen our analysis, and improve the clarity of our text. Both reviewers point out that we were missing Genbank IDs for the reference genomes we used. We include several changes as a response to reviewer concerns –

1) Two additional paragraphs in the discussion section describing the limitations of our study and the rationale behind some of the study design choices.
2) Two additional figures in the supplement describing the N50 of scaffolded assemblies.
3) The Genbank IDs for the reference genomes used in the study.
4) Two additional citations.
5) Additional description in a supplement figure caption.

Reviewer comments are in blue; our responses in black, and new changes to the manuscript in orange.

**Reviewer #1 comment:** The theme of the study is of great importance, but the authors could not soundly design the study. In short, the setting of using each program needs to be optimized for every single case, depending on the genome size of the species of interest, the sequence length distribution of input contig sequences, and the choice of the restriction enzymes used in Hi-C library preparation, and without this kind of optimization, performances of different programs cannot be compared, for the purpose of concluding which program performs better or worse.

**Response to reviewer comment:** We thank the reviewer for their insight, and indeed, the decision on whether to optimize scaffolders is an important one. At first glance, a fair benchmarking approach would be to optimize each parameter in a basic grid search pattern. However, of the five scaffolders we tested, only Lachesis and AllHiC had meaningful optimizable parameters.

1. HiRise does not have optimizable parameters.
2. The main command of 3d-dna does not expose optimizable parameters. While it is possible to specify specific parameters for the submodules 3d-dna uses to run, the authors have this to say about the parameters:

“Scenarios in which modifications of default parameters are typically required are: when working with small amounts of Hi-C data (<7X coverage) or with data generated from a low frequency restriction enzyme, when the Hi-C signal displays strong coverage biases or is noisy such as when working from in dilution Hi-C libraries or libraries from degraded samples, when assembling draft scaffolds with large gaps or badly polished long-read data, when assembling small genomes or drafts that contain large amounts of duplicated sequence due to under collapsed heterozygosity, or when assembling from a partially diploid draft. These problems usually manifest as too much sequence being annotated as ‘debris’ and removed from the assembly process and can be addressed by tuning the edit module (see some comments on this below).

Note that the use of Juicebox Assembly Tools in the majority of cases alleviates the need to perform parameter sweeps on these with defaults working extremely well, requiring only very limited amount of polishing in Juicebox Assembly Tools. And even if the specific assembly project qualifies as one of the ‘special’ non-default cases such as those listed above, 3D-DNA provides output files that can be visually explored in Juicebox Assembly Tools to help with the parameter tuning. See below for a detailed discussion of output files.”

Since we chose to work with high coverage datasets (> 100x) from high quality samples using state-of-the-art assembly methods, we felt that this placed us outside the subset of assemblies where the authors indicated optimization would be useful. We do note that the 3d-dna workflow strongly relies on the Juicebox Assembly Tools (JBAT), which is a manual intervention step. Because our study focused on the algorithmic accuracy of scaffolders, we did not use JBAT for correction. We believe that this exposes the heavy reliance of the method on manual correction, since the tool does not perform as strongly as other methods despite remaining one of the most popular methods. As such, the expectation when using 3d-dna should generally be that each dataset will require several hours of manual correction.

3. Salsa2 does not have optimizable parameters. Users could previously control the number of iterations, but that functionality was deprecated in 2019.
4. Lachesis exposes five parameters for optimization: CLUSTER_MIN_RE_SITES, CLUSTER_MAX_LINK_DENSITY, CLUSTER_DO_NONINFORMATIVE, ORDER_MIN_N_RES_IN_TRUNK, and ORDER_MIN_N_RES_IN_SHREDS. The authors suggest that optimization of these parameters may lead to better results, and indeed, our own experience suggests that tuning these parameters can lead to better results. We discuss our decision not to tune these parameters below.
5. AllHiC appears to utilize many of the same concepts as Lachesis and also exposes a similar set of four optimizable parameters. We discuss our decision not to tune these parameters below.

Given that only two of the five scaffolders had optimizable parameters that the authors suggested tuning, we ultimately opted not to tune any scaffolder. Our rationale was two-fold. First, we felt that it would be a less fair comparison to benchmark untuned scaffolders against tuned scaffolders. If we had tuned Lachesis and AllHiC and found that they performed the best under tuned conditions, this result would suggest that researchers should spend time performing a grid search on the five parameters before they might be able to achieve optimal results. This leads to our second rationale: we felt that the best comparison would be under default and out-of-the box conditions, because that is the most common condition under which software is run, especially in situations where there is no guidance on *how* to optimize the parameters beyond an expensive grid search. Indeed, neither Lachesis nor AllHiC suggest when or in which direction to optimize their parameters.

Our results suggest that Lachesis is a strong contender as one of the best Hi-C scaffolders. Since Lachesis does contain tunable parameters, and our empirical work suggests that results can improve by optimizing them, we agree that parameter tuning merits study. However, we believe that this investigation falls outside the scope of our work, and perhaps could be the focus of a follow-up study that attempts to optimize and streamline HiRise and Lachesis into more modern and user-friendly software tools.

We amended our discussion to add the following paragraph to address these points –

Here, we discuss several key limitations of this study. Our empirical work shows that Lachesis can benefit from optimization of its parameters, but in this work, we chose not to optimize any of the scaffolders for two reasons. First, scaffolders, as with most software, are commonly run using the default parameters. Users may reasonably expect that that tools ought to run well with the default settings the majority of the time, and parameters can be tuned when troubleshooting is necessary. Second, many of the scaffolders did not have optimizable parameters, or the authors cautioned against changing the default settings. For example, we found that HiRise and Salsa2 did not have meaningful parameters to tune, and the authors of 3d-dna suggest running the default parameters in most cases, especially when enough sequencing data is available, and to fix errors manually in Juicebox. Lachesis and AllHiC expose several similar tunable parameters, but do not provide guidance for how to change them and under what circumstances they should be changed. This suggests that a costly grid search might be the best way to explore these parameters.

**Reviewer #1 Comment:** Another caveat is that testing the program's scaffolding performance using split sequences into a uniform length is too far from realism. Normally, there is a huge variation of the continuity of input sequences, which is related to the distribution of difficult regions with high repeat density and GC content.

**Response to Reviewer Comment:** We felt that scaffolders should be tested under two broad categories: the best-case scenario and a real-life scenario. Our best-case scenario involved taking high-quality reference genomes and splitting them into evenly sized pieces to uncover any size-based limitations of a scaffolder. We agree that this scenario does not cover realistic assemblies, and we therefore included our second category of real-life scenarios. For these, we downloaded publicly available long read datasets and performed the assemblies ourselves. We felt this was as close to a real experiment as we could get, and we even downsampled the number of reads to replicate the effect of sequencing depth. Under these circumstances, we show that there is great effect on the quality of the assembly based on the underlying GC content of the species and amount of available sequencing. We generated approximately 11 assemblies for each species under these “real-life” conditions and felt that these sufficiently represented assemblies which groups might encounter in their own assembly work.

**Reviewer #1 Comment:** The authors used the 'known' existing reference genome assembly (without specifying them in particular) as a ground for quantifying the accuracy in scaffolding, but this is not justified as a single metric (although divided into orientation accuracy and ordering accuracy) for the benchmark, because the reference assemblies may contain mistakes.

**Response to Reviewer Comment:** In order to benchmark Hi-C scaffolders, there is a fundamental need for some sort of ground truth against which scaffolding results can be compared. While a synthetic *in silico* genome could be used, we felt that the real-life distribution and content of repeat regions, GC bias, and low complexity regions would be far too challenging to accurately represent. Instead, we opted for a diverse set of high-quality reference genomes.

As the reviewer points out, the exact versions and files for the reference genomes were not provided, and we thank them for pointing out this error. We now provide this table and have amended the manuscript to reflect the Genbank accession IDs for these references.

| Species | Genbank ID |
| --- | --- |
| *S. cerevisiae* | GCA_000146045.2 |
| *L. tarentolae* | Processing |
| *A. thaliana* | GCA_000001735.2 |
| *H. sapiens* | GCA_000001405.28 |

To generate the split assemblies, we partitioned the established reference genomes of *S. cerevisiae* (GCA_000146045.2), *L. tarentolae* (processing), *A. thaliana* (GCA_000001735.2), and *H. sapiens* (GCA_000001405.28) into equal sized pieces of 10kb, 50kb, 100kb, 500kb, and 1mb to create a total of sixteen split reference assemblies.

While these reference genomes may contain errors, we believe that their use is justified for two reasons. First, as model organisms, they have a long history of study, and much effort has gone into the assembly of their genomes. Here, we note that *L. tarentolae* is a well-known and well assembled genome in the parasitology community. Second, we manually inspected each of the four reference genomes using the Hi-C matrices, much as one would do determine if a genome assembly contains any errors. During this inspection, we did not find any regions of concern, suggesting that there are not large scale misassembles present in these genomes.

Finally, the reviewer points out that using the single metric of accuracy is not sufficient to benchmark Hi-C scaffolders. While we believe that accuracy is the most interesting value to researchers, who would opt to increase accuracy over other metrics such as N50 or runtime, our original manuscript includes several other metrics in the supplement. We analyze the grouping accuracy – the ability to separate contigs into their appropriate chromosome, the ordering accuracy – the ability to place a contig next to its expected neighbors, and runtime – the amount of time it takes for a scaffolder to finish running. In addition, in our resubmission we include the scaffolding N50 – the length of contigs at which 50% of the assembly is represented. With these metrics present in addition to accuracy, we believe there should be a sufficient breadth of evidence to draw conclusions as to the performance of Hi-C scaffolders.

**Reviewer #1 Comment:** To improve the entire design of the study, I recommend the authors to refer to the literature included below and more cited therein.

https://genomebiology.biomedcentral.com/articles/10.1186/s13059-022-02765-0
https://onlinelibrary.wiley.com/doi/10.1111/mec.16146
<https://www.nature.com/articles/s41586-021-03451-0>

**Response to Reviewer Comment:** We thank the reviewer for their suggestions on relevant literature for experimental design.

The first paper (Kim, Juwan, et al.) explores the assembly improvements the Vertebrate Genome Project (VGP) has contributed. To this end, they compare the previously available draft genomes of various species to the VGP version. They provide valuable insights into the limitations of short read assemblers and the types of errors they are prone to making.

The second paper (Yamaguchi, Kazuaki, et al.) describes the Hi-C experiment and its applications in genome scaffolding. They go on to describe the various experimental and computational options available during the process. To demonstrate, they perform a genome assembly of the Madagascar ground gecko followed by Hi-C scaffolding to produce a much higher quality genome than previously available.

The third paper (Rhie, Arang, et al.) describes the Vertebrate Genome Project (VGP) and the initial results of the consortium. The VGP has been an insightful and trailblazing association of researchers in the field and have pushed the boundaries of genome assembly. We were aware of their work while completing our study, and we incorporated several sections of their pipeline and methodology into our own work. Chiefly, removing haplotypes from the initial genome assemblies produces much higher quality references, and prevents confusing scenarios for scaffolders.

We add the following citations to our manuscript –

[19] Rhie, Arang, et al. "Towards complete and error-free genome assemblies of all vertebrate species." *Nature* 592.7856 (2021): 737-746.

[20] Yamaguchi, Kazuaki, et al. "Technical considerations in Hi‐C scaffolding and evaluation of chromosome‐scale genome assemblies." *Molecular Ecology* 30.23 (2021): 5923-5934.

**Reviewer #2 Comment:** The authors only tested scaffolding accuracy and speed of the scaffolders, however scaffolding speed is not very well explained in the text, while scaffold N50 is not measured.

**Response to Reviewer Comment:** We felt that in the main text of the manuscript, it was important not to inundate the reader with a huge variety of metrics. Ultimately, we believe this body of work serves as guide to choosing the best Hi-C scaffolder, and researchers would be most interested in the accuracy of a scaffolder over other metrics.

Nevertheless, we include grouping accuracy and ordering accuracy as additional measures of correctness. In addition, since several scaffolders have extraordinarily long runtimes when working on large assemblies, we felt that it would be of interest to estimate how long a given scaffolding run might take. To address the concern of runtime not being sufficiently explained, we have added additional description to the relevant figure caption in the supplement.

**Supplementary Figure 8**. The runtime of Hi-C scaffolders on split assemblies. The runtime refers to the amount of time it takes for a given scaffolding run to finish, given the same amount of computational resources.

While we did collect and plot scaffolding N50, we did not include the figures in our previous draft of the manuscript. We thank the reviewer for pointing out interest in those figures, which we have added in our resubmission, and share below.

**Supplementary Figure 6:** The scaffolding N50 vs the assembly N50 for the split reference assemblies. We see that 3d-dna and AllHiC are prone to “over-scaffolding”, where a far greater than expected number of contigs are joined together, often exceeding even the N50 of the underlying chromosomes.

**Supplementary Figure 7:** The scaffolding N50 vs the assembly N50 for the *de novo* assemblies.

**Reviewer #2 Comment:** The comparison among the scaffolders is not very reasonable, tools like AllHiC and Lachesis require a chromosome number as input, while other not. It's more appropriate to treat them as two groups and perform the comparison.

**Response to Reviewer Comment:** It is indeed the case that several of the most popular Hi-C scaffolders (Lachesis and AllHiC) require chromosome number, while others do not. However, we felt that this was not a sufficient reason to exclude or segregate these scaffolders. We offer three reasons why we chose to include them in our study.

1) The chromosome number is often known. One of the most common settings for Hi-C scaffolding is for reassembling the genomes of previously assembled species using modern sequencing approaches. Many genomes of interest are in the plant and animal kingdoms, and generally have large genomes. While the last generation of short read sequencing methods most often yielded poor assemblies, other characteristics such as GC content, genome size, and chromosome count were commonly identified through either experimental or computational methods.
2) Chromosome count can be estimated. If the chromosome count of the species under study is not known, then the chromosome count of the most closely related species with a well annotated genome can be used as an initial estimate. Generally, after Hi-C scaffolding is complete, it is evident whether chromosomes need to be merged or divided.
3) It is sufficient to make arbitrary chromosome counts. Our experience with Hi-C scaffolders has shown that we can start with an arbitrary chromosome count such as 20 to start the scaffolding process. Most commonly, the chromosome count is used to determine the number of clusters during the initial steps of scaffolding. From the final output of the scaffolder, we can then determine if the number of clusters was too high or too low.

In our resubmission, we include the following text in the discussion section -

Lachesis and AllHiC require chromosome count, a constraint not present for the other scaffolders. The number of chromosomes is not always known, especially in new sequencing endeavors, leading to some uncertainty as to the applicability of these methods in under such circumstances. However, in our empirical work, we found that the chromosome count can be an arbitrary estimate that can be tuned after an initial run to determine if there was over clustering or under clustering of contigs. As such, we believe the five scaffolders tested are broadly interchangeable.

**Reviewer #2 Comment:** More scaffolders should be involved into this test to find the best performer.

**Response to Reviewer Comment:** We performed a literature review to determine available Hi-C scaffolders as illustrated in Table 1. Of the nine scaffolders identified, we found that several had not been used since the time of their publication. To provide the field with the most benefit, we focused on scaffolders with three or more citations where the authors used the method to publish a genome. We added the following paragraph in our discussion section to provide additional context for this decision.

Additionally, we chose not to test all the scaffolders we uncovered during our literature search. Primarily, we wanted to focus our efforts on methods which have been used in the publication a genome, thereby demonstrating the usability of a method. We found that dna-triangulation was positioned more as a proof-of-concept rather than a piece of software intended for use by other groups. In our testing and in correspondence with other groups, we found that GRAAL had a tricky GPU and GUI requirement, which has limited its wider adoption. Hi-C Hiker only determined the orientation of a contig within a given scaffold, and therefore only accomplished a subset of our prescribed task. Finally, HiCAssembler was a potential contender as a sixth method to test, but we felt that adhering to our inclusion/exclusion criteria meant we could not include the scaffolder since it did not have any published papers using it at the time of our literature review.

**Reviewer #2 Comment:** The authors should explain how different N50 assemblies are obtained.

**Response to Reviewer Comment:** We describe how the different N50 assemblies are obtained in the main text and supplement. In the methods section we write:

“To generate the split assemblies, we partitioned the established reference genomes of *S. cerevisiae*, *L. tarentolae*, *A. thaliana*, and *H. sapiens* into equal sized pieces of 10kb, 50kb, 100kb, 500kb, and 1mb to create a total of sixteen split reference assemblies. To generate a diverse set of *de novo* assemblies for each of the four organisms, we collected a large repository of long-read data from NCBI’s SRA database, as well as our previously generated PacBio reads for *L. tarentolae* (see Supplementary Table 3). To normalize for different genome sizes, we downsampled the number of reads to achieve a theoretical coverage of 10x, 20x, …, 100x for each reference genome. We ran the Canu assembler on each of these down-sampled datasets using the default parameters to create forty different *de novo* assemblies summarized in Supplementary Table 4.”

Supplementary Table 4 then describes the relationship between down-sampling long reads and the effect it has on the N50 of the *de novo* assemblies –

| **Coverage** | **Yeast N50** | **Leishmania N50** | **Arabidopsis N50** | **Human N50** |
| --- | --- | --- | --- | --- |
| 10 | 21,825 | 9,250 | 24,823 | 18,711 |
| 20 | 176,516 | 18,595 | 86,731 | 43,231 |
| 30 | 551,752 | 41,537 | 159,879 | 105,563 |
| 40 | 568,123 | 40,610 | 147,845 | 626,886 |
| 50 | 614,056 | 94,577 | 149,418 | 1,873,143 |
| 60 | 813,309 | 213,275 | 161,138 | 2,018,914 |
| 70 | 777,771 | 71,335 | 140,209 | 4,761,131 |
| 80 | 813,629 | 117,224 | 149,909 | 7,508,518 |
| 90 | 813,427 | 229,090 | 164,531 | 9,231,632 |
| 100 | 930,538 | 332,478 | 186,445 | 10,553,285 |

“**Supplementary Table 4.** Overview of the *de novo* assemblies created by Canu. We generated ten assemblies for each species and downsampled reads used to create them to vary their N50s. As a general trend, we observed that increased read coverage led to long contigs. Arabidopsis appeared to be an outlier of this trend, and its genome assembly appeared to be challenging and indicative of high rates of heterozygosity.”

**Reviewer #2 Comment:** The authors should list the genome accessions instead of project ID.

**Response to Reviewer Comment:** We thank the reviewer for pointing out this area of concern. In our resubmission, we specify the Genbank IDs for the reference genomes utilized. We believe that the project IDs are still relevant as they contain information on the SRA runs where we downloaded both long read and Hi-C sequencing data.

To generate the split assemblies, we partitioned the established reference genomes of *S. cerevisiae* (GCA_000146045.2), *L. tarentolae* (processing), *A. thaliana* (GCA_000001735.2), and *H. sapiens* (GCA_000001405.28) into equal sized pieces of 10kb, 50kb, 100kb, 500kb, and 1mb to create a total of sixteen split reference assemblies.

**Reviewer #2 Comment:** We recommend to use T2T assemblies to perform the test, since they should contain fewer errors.

**Response to Reviewer Comment:** It is indeed the case that the T2T human assembly will likely replace the GRCh38 as the de facto standard in genomics. However, we believe that the GRCh38 assembly used in our study is of sufficiently high quality that it would not significantly impact our results. Our manual inspection of the Hi-C matrix using the GRCh38 reference did not show significant regions of misassembly.

Additionally, the most computationally expensive portions of our study are related to the human genomes, since they are the largest datasets. In total, we spent approximately 91 years in CPU hours on the study, and we feel that repeating a substantial portion of the experiment would not yield sufficiently different results to justify the time and cost it would take to complete.

**Reviewer #2 Comment:** The author should mention what coverage should be applied to get more accurate scaffolds for most scaffolders.

**Response to Reviewer Comment:** We discuss the minimum coverage of Hi-C reads required before a degradation in scaffolding is observed in the results section of the main text:

“We found that the vast majority of the time, performance degrades as the amount of Hi-C data is reduced (Figure 1). Although we observe heterogeneity as to when this shift occurs, 50 reads/kilobase appears to be the point below which at least some of the scaffolders begin to perform worse.”

**Second round of review**

**Reviewer 3**

1. I recommend that, at a minimum, they are encouraged to reach out to the authors of each tool for guidance on parameter settings and then make an attempt to run it.

2. Clarify how coverage levels affect scaffold accuracy across different tools and datasets.

3. Additionally, the readers may find it valuable to consider the evaluation metrics discussed in the following resource:
<https://genomebiology.biomedcentral.com/articles/10.1186/s13059-021-02328-9>

4. "However, more surprising is the second trend, where scaffolders also perform worse in the high N50 regime". A small experiment could be conducted to explore whether larger contiguous sequences might reduce the resolution of Hi-C data, making it more challenging to pinpoint precise interactions. One potential direction is to examine unique variant rates among contigs (e.g., see <https://www.nature.com/articles/s41467-023-36689-5> ). Based on these findings, a recommendation could be made to integrate both N50 and unique variants for a more comprehensive evaluation of scaffolder effectiveness.

**Authors Response**

**Point-by-point responses to the reviewers’ comments:**

**Summary:** The reviewer provided several valuable insights on improving the manuscript. They pointed out that we should more deeply explore the optimal parameters with which to run each scaffolder. Additionally there was feedback about improving the clarity with which we discuss the optimal coverage of Hi-C reads. Finally, there was a suggestion to explore additional papers which discuss other methods of assessing scaffolder performance. Here, we discuss this feedback and offer several changes.

Reviewer comments are in blue; our responses in black.

1. I recommend that, at a minimum, they are encouraged to reach out to the authors of each tool for guidance on parameter settings and then make an attempt to run it.

For several of the methods we reach out to the authors for optimal parameters, here was the guidance we received for each one –

a) Lachesis was no longer officially supported, though several collaborators had indicated a grid search of the parameters could lead to better results.
b) 3d-dna, the official guidance we received was that the parameters general did not have that much of an effect on the outcome and that manual correction using Juicebox tools was the preferred method of dealing with ambiguities.
c) Hirise went on to become a private company after the publication of the initial software. Its publicly available software does have any of the underlying parameters exposed and the tutorial runs the software with no flags or parameters besides the underlying files.
d) Salsa only has two available parameters to tune, minimum contig length and iterations. In the portion of the paper evaluating assemblies with fixed contig lengths, they were of a uniform size, and the parameters was not applicable. We did attempt to use the iterations parameter but did not find it caused a noticeable change in outcomes.
e) Allhic did have several possible parameters to tune though the tutorial suggested using the default parameters. -m minimum single density for rescuing contigs (optional, default 0.01), --npop value	Population size (default: 100), --ngen value	Number of generations for convergence (default: 5000), --mutpb value Mutation prob in GA (default: 0.2)

In all the cases, we set the enzyme parameter appropriately as well as the number of chromosomes for those methods that accepted it. Since several of the methods effectively did not have parameters to optimize and two of them did, we made the choice to run all the methods on the default parameters. However, we do acknowledge that running software on its default setting is not always optimal, and several of our colleagues have observed the Lachesis in particular can benefit the outcome. To this end, Lachesis itself provides some troubleshooting as to how to pick parameters. Here are the relevant sections –

**Creating an INI file**

Many of the other INI parameters provide heuristic parameters. It's worth your time to examine these parameters to get a better idea of what LACHESIS is doing. But if you're unsure of any of them, feel free to use the values in INIs/test_case.ini as defaults.

**Running LACHESIS**

There are many parameters in the INI file beyond the inputs to LACHESIS. You may need to tweak some of these parameters in order to get an optimal assembly. Be sure to read the documentation in the INI file carefully so that you know what you're doing.

**LACHESIS is producing a weird result!**

There are several heuristic parameters involved in the running of LACHESIS. They include: CLUSTER_MIN_RE_SITES, CLUSTER_MAX_LINK_DENSITY, CLUSTER_DO_NONINFORMATIVE, ORDER_MIN_N_RES_IN_TRUNK, and ORDER_MIN_N_RES_IN_SHREDS. These parameters are tuning knobs that can be tweaked as necessary to produce a high-quality draft assembly. For the LACHESIS publication, these parameters were set to values that are appropriate for the datasets that went into those assemblies, and these are the values you can see in the INI files that are provided with the LACHESIS distribution. However, these values may not work for your situation. The ideal values depend on the repeat content of the genome, the N50 of your input assembly, your density of Hi-C data, and your preference for accuracy versus completeness.

Try tweaking some of the parameters and re-running LACHESIS. The easiest approach is to produce a good clustering result first, then go from there to ordering (and make sure to set OVERWRITE_CLMS = 1 when you move from clustering to ordering.) If you have a reference genome to compare your result against (USE_REFERENCE = ), the reference-based evaluation will be very helpful.

To address this, we have added the additional context in the discussion –

“Lachesis was the second best scaffolder on average, though it was the tool that most commonly failed to run under default settings. Nevertheless, we found that by tweaking parameters, it is generally possible to improve results, and we would recommend that users perform a grid search to find the optimal setting. Its initial release also contains several installation bugs, and its later development has been taken on by another private company (Phase Genomics).”

“Here, we discuss several key limitations of this study. Our empirical work shows that Lachesis can benefit from optimization of its parameters, but in this work, we chose not to optimize any of the scaffolders for two reasons. First, scaffolders, as with most software, are commonly run using the default parameters. Users may reasonably expect that that tools ought to run well with the default settings the majority of the time, and parameters can be tuned when troubleshooting is necessary. Second, many of the scaffolders did not have optimizable parameters, or the authors cautioned against changing the default settings. For example, we found that HiRise and Salsa2 did not have meaningful parameters to tune, and the authors of 3d-dna suggest running the default parameters in most cases, especially when enough sequencing data is available, and to fix errors manually in Juicebox. Lachesis and AllHiC expose several similar tunable parameters, but do not provide guidance for how to change them and under what circumstances they should be changed. This suggests that a costly grid search might be the best way to explore these parameters.”

2. Clarify how coverage levels affect scaffold accuracy across different tools and datasets.

This is indeed an important finding in the paper, we have revised the manuscript to more prominently feature these results in the discussion as well.

**Results**

**Figure 1.** The effect of down-sampling Hi-C coverage on scaffolding accuracy. While using the 100kb N50 split reference assembly, reads were down sampled to target densities. Past 50 reads per kilobase, performance of all scaffolders tends to degrade, though some scaffolders are more resistant to decline than others.

“Although we observe heterogeneity as to when this shift occurs, 50 reads/kilobase appears to be the point below which at least some of the scaffolders begin to perform worse.”

**Discussion**

“We found that a minimum of 50 aligned reads per kilobase is the threshold required before scaffolding performance degrades, providing guidance on the lower limit of Hi-C sequencing based on assembly size.”

3. Additionally, the readers may find it valuable to consider the evaluation metrics discussed in the following resource:
<https://genomebiology.biomedcentral.com/articles/10.1186/s13059-021-02328-9>

We thank the reviewer for this article examining computational methods for reconstructing chromosome-scale haplotypes. The article traces the evolution from short-read sequencing technologies that cannot produce whole-chromosome haplotypes to third-generation long-read technologies (PacBio, Oxford Nanopore) and chromosome-scale methods (Hi-C, Strand-seq) that enable comprehensive haplotype reconstruction. Three main computational approaches are discussed: reference-based phasing using tools like WhatsHap and HapCut2, de novo haplotype assembly through collapsed/semi-collapsed/uncollapsed strategies, and strain-resolved metagenome assembly for microbial communities, with specialized methods required for polyploid genomes containing multiple haplotypes. The review highlights key evaluation metrics including switch error rates, Hamming error rates, phasing completenes, haplotype block N50 lengths, and base quality scores. Additionally the article references several well known assembly competitions; CAMI, Assemblathon, and GAGE. These three studies greatly informed our study design, and we found the discussion section of these papers invaluable. In particular, this section of Aseemblathon 2 resonated with us -

**Lessons learned from assemblathon 2**

The clear take-home message from this exercise is the lack of consistency between assemblies in terms of interspecific as well as intraspecific comparisons. An assembler may produce an excellent assembly when judged by one approach, but a much poorer assembly when judged by another. The SGA snake assembly ranked 1st overall, but only ranked 1st in one individual key metric, and ranked 5th and 7th in others. Even when an assembler performs well across a range of metrics in one species, it is no guarantee that this assembler will work as well with a different genome. The BCM-HGSC team produced the top ranking assembly for bird and fish, but a much lower-ranked assembly for snake. Comparisons between the performance of the same assembler in different species are confounded by the different nature of the input sequence data that was provided for each species.

To this end we decided to make two choices – first, to focus on scaffolding specific metrics, and second, to limit the number of metrics we evaluated the assemblies by. By focusing on scaffolding metrics, we explicitly assume that the underlying assembly is correct. To achieve this, we utilized four pre-existing high quality assemblies and then broke them up into constituent contigs. This allowed us to avoid issues of base or region quality, collapsed repeats, and other assembly specific issues. On the second point, in the primary paper we exclusively utilize scaffolding accuracy which we have defined using the edit distance required to “fix” the genome. We discuss these metrics further in the next section.

4. "However, more surprising is the second trend, where scaffolders also perform worse in the high N50 regime". A small experiment could be conducted to explore whether larger contiguous sequences might reduce the resolution of Hi-C data, making it more challenging to pinpoint precise interactions. One potential direction is to examine unique variant rates among contigs (e.g., see https://www.nature.com/articles/s41467-023-36689-5). Based on these findings, a recommendation could be made to integrate both N50 and unique variants for a more comprehensive evaluation of scaffolder effectiveness.

We agree with the reviewer that uncovering why scaffolders perform worse with higher N50 assemblies is an important point for the paper. In our experimentation, we found that the primary reason for this was over clustering, i.e. not separating legitimate chromosomes from each other. Luckily this is an easy error to spot manually using the Juicer suite of tools. To illustrate this, we include Supplementary Figure 12.

**Supplementary Figure 12.** AllHiC scaffolding 500kb contigs from the split reference assembly of *S*. *cerevisiae.* While the contigs have been placed mostly in the correct order and orientation, all the contigs were placed in a single mega-scaffold causing the overall accuracy to dramatically decrease for this particular scaffolding.

The reviewer also alludes to the fact that sequencing uniqueness may affect the ability to scaffolding using Hi-C. We did indeed notice this decrease in scaffolding performance with more repetitive genomes such as *A. thaliana*. There are two aspects which make low complexity regions challenging to scaffold, haplotigs and repeats. In the case of haplotigs, the Hi-C scaffolders we analyzed did not have the ability to place two haplotypes in the same genomic region, but rather one after another, which leads to downstream scaffolding errors.

**Supplementary Figure 11.** An overview of how HiRise scaffolded the 10mb N50 *H. sapiens* assembly for Chromosome 22. Each row represents a contig, and each label and color represents a scaffold. The x-axis represents the alignment based position of the contig, and the y-axis represents the scaffolder based order of the contigs. Here, HiRise picks out a set of larger contigs and scaffolds them in the correct order relative to each other. However it leaves out a number of the smaller contigs, including ones that overlap with its primary scaffold (Scaffold 503) for this chromosome.

In the case of repeats, Hi-C reads are not able to be uniquely placed and therefore are lost in the processing pipeline. In highly repetitive genomes, this can lead to a dramatic loss in coverage across certain contigs. To highlight this, we include Supplementary Figure 14.

**Supplementary** **Figure 14.** The percent of reads that map to multiple positions in the *de novo* assembly. We found that as the number of reads used to create the *de novo* assembly goes up, the repetitive content of the genome goes up. The uniformly low accuracy against *A. thaliana* assemblies can likely be attributed to a high percentage of multi-mapping reads, which cannot be used by Hi-C scaffolders.

In addition, we include the following section to discuss the impacts of repetitive regions in the main text -

“While investigating the lower performance on the *de novo* assembly of *A. thaliana*, we discovered that approximately fifty percent of the Hi-C reads for the *A. thaliana* assemblies mapped to more than one location, in comparison to about ten percent for the species (Supplementary Figure 14). Because multi-mapping reads cannot be reliably used in the scaffolding process, Hi-C scaffolders typically mask these regions during preprocessing. We speculate that this led to the poor scaffolding performance on *A. thaliana* assemblies (Supplementary Table 3). For such repetitive genomes, we speculate that more traditional methods such as optical mapping or new methods to span repeats such as ultra-long nanopore reads may be required.”

Finally, on the topic of which metrics to utilize for the paper, we rely on our previous work using Edision which finds the minimum number of edits required to correct the scaffolds into the ground truth (https://www.biorxiv.org/content/10.1101/2022.03.25.484952v1.full). In particular, we utilize the accuracy measurement from this method which is conceptually similar to the percent of bases that don’t have to be moved while fixing the genome. In the Edison paper, we explore N50 versus the scaffolding accuracy measurement and find that while accuracy retains our sense of which scaffolds are accuracy and which are not, N50 can be gamed by merging chromosomes together.
